# Developing and evaluating an intervention to improve the disposal of compostable packaging at UK workplaces

**DOI:** 10.1177/0734242X251322145

**Published:** 2025-02-26

**Authors:** Nicola J Buckland, Sara Bru Garcia, Rosie Sharp, Tom Mockridge, Sarah Greenwood, Meghann Matthews, Thomas L Webb

**Affiliations:** 1 School of Psychology, University of Sheffield, Sheffield, UK; 2 Hubbub, London, UK; 3 Recorra, London, UK; 4 Department of Chemistry, University of Sheffield, Sheffield, UK; 5 Grantham Centre for Sustainable Futures, University of Sheffield, Sheffield, UK

**Keywords:** Compostable packaging, behaviour change intervention, waste behaviours, sustainability, COM-B model, Behaviour Change Wheel

## Abstract

Compostable packaging may provide a more sustainable alternative to conventional packaging. However, people often inappropriately dispose of compostable packaging which limits its potential benefits. This study applied the Behaviour Change Wheel and the capability, opportunity, motivation-behaviour model to develop and evaluate the effect of a behaviour change intervention on the disposal of compostable packaging at workplaces. Focus groups, observations, analysis of existing materials and a review of the literature identified barriers in relation to capability, opportunity and motivation to compost packaging. The intervention addressed the identified barriers through clear and distinctive labels on packaging, matching bin signage, a motivational video and an onboarding presentation for workplaces. The intervention was implemented in three workplaces and evaluated using a pre–post design. The intervention led to sustained increases in compostable packaging in compostable bins and reduced contamination. A post-intervention survey and roundtable event suggested that the intervention was acceptable to workplace leads and employees. There was no evidence that the intervention changed perceived capability, opportunity and/or motivation. The main recommendations for packaging producers and workplaces include using consistent distinctive bin signage that corresponds with standardized packaging labels.

## Introduction

The environmental and health risks of plastic waste necessitate alternative solutions (Plastics Europe, 2022; Rist et al., 2018). The food service industry widely uses single-use, non-degradable plastics to package food and drinks. However, there is a growing industry using certified compostable plastics (Packaging World, 2023). When composted under controlled conditions with food or garden waste (e.g. in an industrial composting facility), certified compostable packaging breaks down into water, carbon dioxide and compost to offer a potentially more sustainable alternative to conventional plastics.

Yet, the environmental benefits of compostable packaging depend on consumers’ correctly disposing of compostable packaging and not contaminating the waste stream with conventional plastics or other non-compostable materials. Unfortunately, evidence suggests that consumers often inappropriately dispose of compostable packaging. For example, most participants disposed of packaging labelled as ‘compostable’ in the recycling rather than organics bin (Ansink et al., 2022; Taufik et al., 2020) and 28% of survey respondents said they would put compostable packaging in the recycling bin (Closed Loop Partners’ Composting Consortium & Biodegradable Products Institute, 2023). Therefore, consumers need to be supported and prompted to put compostable packaging into the correct bin for industrial composting.

A potential barrier to the correct disposal of compostable packaging is low awareness of compostable packaging and where to dispose of it (Allison et al., 2021). Such confusion occurs despite consumers expressing preferences for more sustainable packaging formats (Statista, 2022), positive attitudes towards compostable packaging (Koenig-Lewis et al., 2022; Norton et al., 2022) and reporting intentions to purchase compostable packaging (Allison et al., 2021; Granato et al., 2022; Koenig-Lewis et al., 2022). Therefore, there is an attitude–behaviour gap (Kollmuss and Agyeman, 2002); people express positive attitudes towards compostable packaging, however, do not act in environmentally beneficial ways.

Behaviour change interventions that target the determinants of this attitude–behaviour gap have the potential to help people correctly dispose of compostable packaging. Systematic reviews of interventions designed to promote home composting and recycling report favourable effects of interventions, especially those that involve restructuring the physical environment (e.g. increasing the number or proximity of bins) and social modelling (Sewak et al., 2021; Varotto and Spagnolli, 2017). A meta-analysis of 33 interventions targeting the reduction of plastic waste also reported positive effects ranging from small to very large (Allison et al., 2022a). To date, however, no interventions have focused on promoting the appropriate disposal of compostable material for industrial composting.

Theory-driven interventions which target the barriers underlying the attitude–behaviour gap are more likely to change behaviour than less targeted interventions (Michie et al., 2008). The Behaviour Change Wheel (BCW) provides a theoretical framework for designing and evaluating interventions and was developed following a review of nineteen frameworks and initial examination of reliability in two domains of behaviour change: tobacco control and obesity (Michie et al., 2011). Since 2011, the BCW has been used to develop a wide range of interventions in a wide range of contexts, including in relation to reducing plastic waste (for a review, see Allison et al., 2022a), where it is recommended as a way to understand behaviour and design interventions (MacDonald et al., 2023).

The BCW outlines two main stages to intervention development. Firstly, defining the problem in behavioural terms, understanding current behaviour, and what needs to change to result in the desired behaviour. ‘What needs to change’ is specified using the COM-B model which identifies capability, opportunity and motivation as drivers of behaviour. Capability consists of physical (e.g. physical strength, stamina) and psychological capability (e.g. knowledge). Opportunity consists of physical (e.g. that appropriate bins are available) and social opportunity (e.g. that important others approve of the behaviour). Motivation refers to the drive to engage in the behaviour and consists of automatic (e.g. emotional and unconscious processes) and reflective motivation (e.g. beliefs about the beneficial consequences). For a behaviour to be enacted, the COM-B model suggests that all components need to be in place.^1^ The second stage involves identifying the potential intervention options that may be effective. The BCW presents nine potential intervention functions linked to policy options. Intervention functions and/or identified barriers/enablers in capability, opportunity and motivation can then be mapped onto behaviour change techniques (BCTs) to deliver as part of the intervention content (Marques et al., 2023; Michie et al., 2013).

Using the BCW to improve households’ disposal of compostable packaging, Allison et al. (2022b) recommended adding a label on compostable packaging with disposal instructions. However, such labelling recommendations are yet to be evaluated, and there is limited systematic behavioural analysis to identify what needs to change and be targeted within an intervention. As such, further research is needed to apply the BCW to develop *and* evaluate an intervention to improve the disposal of compostable packaging. Furthermore, Allison et al.’s (2022b) recommendations focused on the disposal of compostable packaging within UK households; however, compostable packaging is used in other contexts, such as within closed loop systems.

Closed loop systems are those where compostable packaging is circulated and collected on-site for industrial composting (e.g. workplace canteens, festivals; WRAP, 2020). Closed loops are a potential useful application of compostable packaging because the variety of packaging formats and bins available can be limited and controlled to support the appropriate disposal of compostable packaging.^2^ Crucially, the benefit of using compostable packaging within closed loops depends on people appropriately disposing of compostable packaging. Consequently, closed loop contexts are a priority to evaluate the effectiveness of interventions to encourage the appropriate disposal of compostable packaging.

This research aimed to develop and test the effects of an intervention on the amount of compostable packaging composted in closed loop contexts; namely, UK workplaces. The study also aimed to test the effect of the intervention on putative determinants of behaviour – perceived capability, opportunity and motivation. The specific objectives were to: (i) understand beliefs and behaviours related to compostable packaging to identify which COM-B components to target for change; (ii) develop an intervention and (iii) evaluate the effects of the intervention on the amount of compostable packaging composted at workplaces and perceived capability, opportunity and motivation to compost among employees. It was expected that compared to pre-intervention, there would be a greater amount (%) of compostable packaging in compostable bins at post-intervention and follow-up. This increase was expected to correspond with reduced contamination in compostable bins at post-intervention and follow-up compared to pre-intervention. It was also expected that there would be less compostable packaging in other waste streams (dry mixed recycling (DMR) and general waste) at post-intervention and follow-up compared to pre-intervention. Secondary hypotheses predicted increases in perceived capability, opportunity and motivation to identify and appropriately dispose of compostable packaging at post-intervention compared to pre-intervention.

## Methods

### Design

The research involved three stages: a behavioural analysis to understand current behaviours and identify which COM-B components to target to improve composting. This involved assessing existing materials designed to support effective composting in workplaces, on-site observations, focus groups and consulting previous literature.^3^ Stage 2 applied the BCW and COM-B model to develop an intervention to address identified barriers. Finally, the impact of the intervention on waste behaviour (primary outcome) and perceived COM-B components (secondary outcomes) was evaluated. A roundtable event with workplaces and project partners assessed acceptability and experiences of delivering and evaluating the intervention.

The workplaces invited to take part operated within a closed loop, whereby compostable packaging was supplied by Vegware and waste was collected by Recorra. Twenty-two workplaces were invited. Six agreed to take part. One workplace withdrew before the observations and focus groups were conducted. Five workplaces took part in the observations. Four took part in the focus groups and the intervention. One workplace withdrew before the intervention was implemented, resulting in three workplaces who completed the intervention (workplaces A, B and C^4^). The workplaces consisted of a legal, insurance and property firm with staff offices of around 100, 750 and 1000 employees.

Ethical approval was obtained from the University of Sheffield ethics committee (approval no. 049479; 051219; 056685). Focus group and survey respondents provided informed consent prior to participation. No informed consent was obtained from staff in relation to waste audit as no individuals were specifically recruited for this part of the study. Protocols were pre-registered on the Open Science Framework (https://osf.io/xfgwj/).^5^ The intervention was designed based on the BCW and Behaviour Change Taxonomy v1 (Michie et al., 2013). For interoperability cross-references to the Behaviour Change Technique and Intervention Ontology (BCIO; Marques et al., 2023) will be reported where relevant.^6^

### Stage 1: Behavioural analysis

#### Target behaviours, setting and population

The behaviours to target were selected based on discussions with project partners and a review of existing materials provided to workplaces by Vegware. The selected behaviours were: (i) putting compostable packaging into compostable bins and (ii) avoiding putting contaminating materials into compostable bins. These behaviours took place within commercial office workplaces (BCIO:026037) in England, UK where compostable packaging and compostable bins were available within closed loops (e.g. in canteens) for use by employees while at work (BCIO:036110). Employees comprised mostly of office staff using the canteen and kitchen and catering staff.

#### Existing materials

Two researchers (NJB, TLW) coded 18 materials that Vegware offered to workplaces using their packaging, including bin signage, posters and a motivational video (see Supplemental Table S1).^7^ The purpose of coding the materials was to identify: (i) potential behaviours to target, (ii) potential COM-B components to target and (iii) whether workplaces implemented the materials as intended. This latter question was investigated via the observations and focus groups conducted at workplaces. For each material, the following was coded: target behaviours (e.g. identifying/checking packaging type, putting compostable packaging in the correct bin), target populations (e.g. retail/cleaning staff, customers), targeted COM-B and theoretical domains framework component(s) (Cane et al., 2012), BCTs (using the BCT Taxonomy v1; Michie et al., 2013, 2014), intervention function and mode of delivery (Marques et al., 2020).

The coding process and resulting data is in Supplemental Table S2. The materials targeted each COM-B component (see Supplemental Table S2). The environmental consequences of composting were frequently referred to, but there was limited reference to the personal consequences of composting. As such, highlighting the personal relevance of composting and targeting emotions was identified as a potential area to target (automatic and reflective motivation).

#### Focus groups

Seven focus groups with 29 kitchen, cleaning or office staff were conducted at 4 workplaces.^8^ A discussion guide assessed perceived capability, opportunity and motivation to identify compostable packaging and put compostable packaging in compostable bins (https://osf.io/xtwrs). Thematic analysis (Braun & Clarke, 2006) identified themes which were mapped onto the COM-B components^9^ (see Supplemental Table S3).

A main barrier identified was confusion identifying compostable packaging as participants reported that it looked like conventional packaging (psychological capability). Participants also reported difficulty sorting waste due to unclear bin signage and multiple bins available (psychological capability). Another barrier was that the consequences of composting were not immediately obvious or relatable (reflective motivation). Participants also indicated that existing materials (see section ‘Existing materials’) were not being implemented at workplaces (physical opportunity).

#### Observations

Visits to observe workplaces were undertaken by Hubbub^10^ who observed compostable packaging, bins (e.g. layout, look of bins), bin signage, instructions about packaging disposal and any communications related to compostable packaging. A main barrier identified was that there were multiple packaging formats available (e.g. recyclable, compostable), but no clear distinction between packaging formats^11^ (psychological capability). Similarly, there were different brands of compostable packaging with varying packaging designs and messages. A second barrier was that there were multiple bins, and it was unclear which items should go in each bin as compostable bins were not clearly marked (psychological capability). Another barrier was that there was no evidence that the existing materials offered to workplaces were being implemented (physical opportunity). As such, the signage was inconsistent, as were the messages about compostable packaging across the workplaces. Additionally, one workplace only had a compostable bin in offices and not the canteen^12^ (physical opportunity).

### Stage 2: Intervention development

The University of Sheffield and Hubbub undertook meetings whereby the barriers identified in stage 1 were mapped onto the COM-B components^13^ and intervention ideas to address each barrier were generated. Intervention ideas were shared with partners (Recorra and Vegware) to shortlist intervention components to develop for implementation. Intervention components were refined for development and implementation.^14^ For resulting intervention components mapped to COM-B components, intervention functions, BCTs and mode of delivery, see Supplemental Table S4.

**Figure 1.**
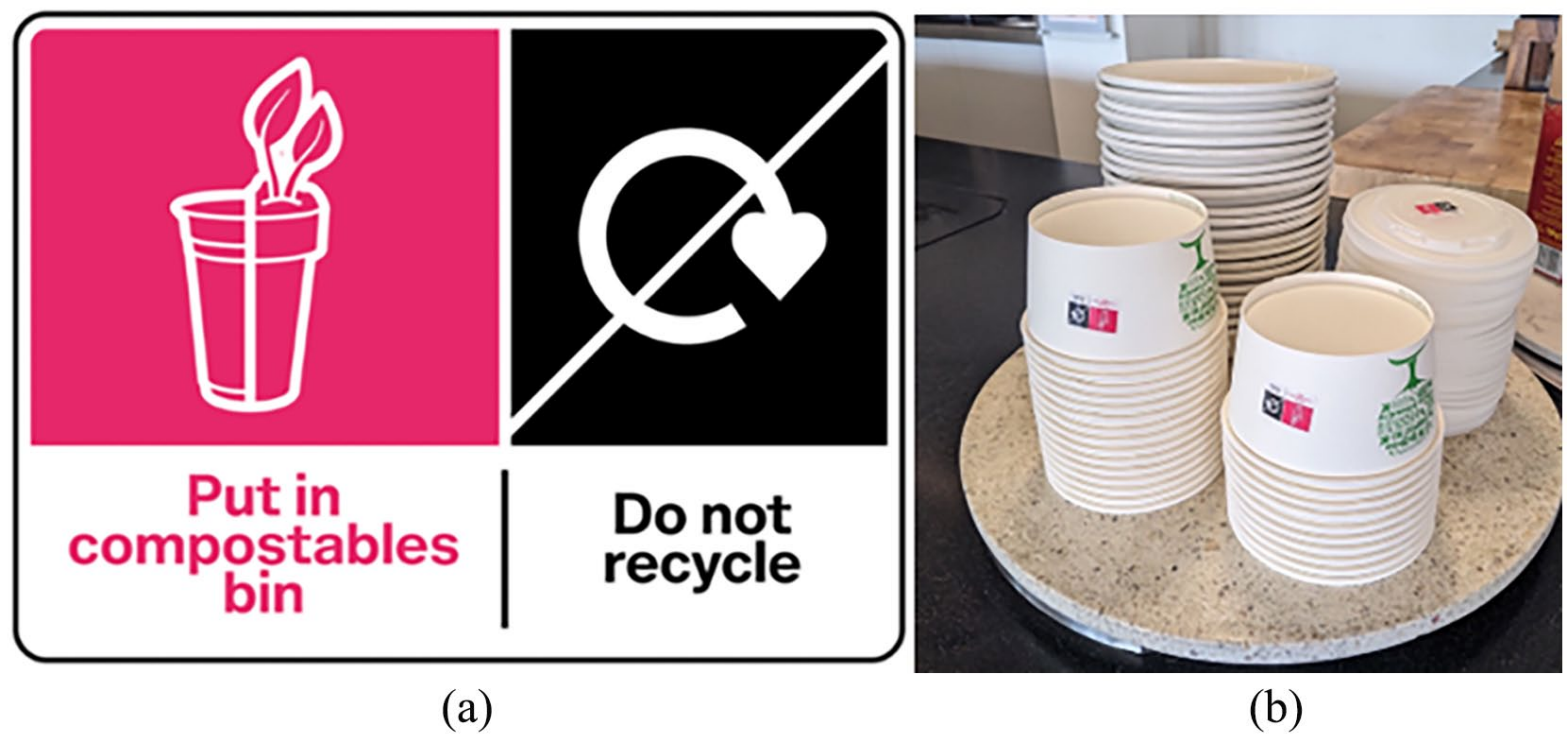
Label developed (a) which was applied to compostable packaging (b).

Two intervention components were developed to address confusion identifying compostable packaging and sorting waste (psychological capability). Firstly, a visually salient and distinctive pink label with a logo with disposal instructions was applied to compostable packaging at workplaces (see Figure 1). The use of a logo and text was informed in part by previous work (https://osf.io/vj9hy/). The colour pink was selected due to its saliency and distinctiveness given that no other UK packaging disposal labels are pink (high distinctiveness). Hubbub designed the label with input from On-Pack Recycling Labels.^15^ The label featured a logo displaying a plant with a base appearing to be half a coffee cup and half a plant pot. This design was selected from three options based on team discussions and market research conducted with Hubbub.^16^ The compostable label was displayed as part of a dual-label which included the United Kingdom’s ‘Do not recycle’ label to ensure that the label was consistent with upcoming labelling legislation (Letsrecycle.com, 2023). Labels were applied as stickers^17^ to all compostable packaging available at each workplace to reflect labels integrated into packaging design.

**Figure 2.**
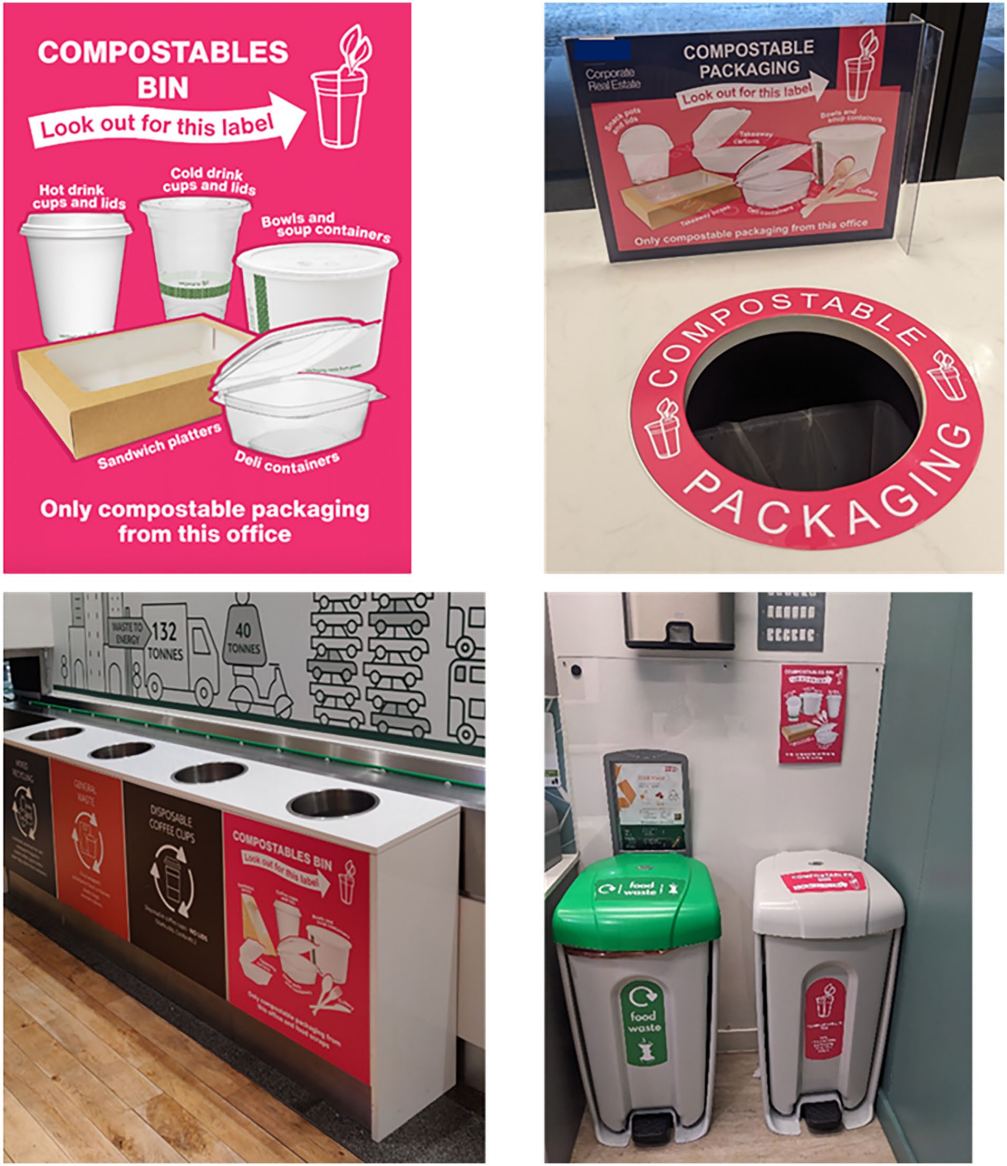
Signs installed at workplaces.

Secondly, signs were installed on and near compostable bins at workplaces (see Figure 2). Pink signs were fitted to bins for compostable waste and labelled as ‘Compostable bins’. The signs indicated which packaging could be put into compostable bins, referred to the compostable label (‘Look out for this label’) and disposal instructions (note, two workplaces encouraged no food in compostable bins, one workplace allowed for food scraps). The signs were colour-matched to the pink label to prompt people to put the packaging in the corresponding bins.

Thirdly, a video aimed at increasing motivation to put compostable packaging in compostable bins was produced by Vegware to be shared at workplaces (adapted version here: https://brandfolder.com/s/6s54t4zr54xjw6f2qw9nv2t^18^). The video explained what compostable packaging is and showed someone demonstrating putting compostable packaging in a compostable bin. The video explained that, when industrially composted, compostable packaging breaks down with food and garden waste to create compost which ‘feeds fields’, and in turn, ‘fields feed us’ – the wording and scene (someone eating a sandwich) were selected to highlight the relatable consequences of composting.

Fourthly, to address the barrier that workplaces were not implementing existing materials as intended (physical opportunity), an on-boarding presentation was developed and delivered by behavioural scientists (SBG, NJB, TLW) to workplace leads to encourage them to deliver the intervention as intended (see https://osf.io/p8zrc).

Finally, in addition to the main intervention components, two compostable bins were added to the canteen in workplace A as prior to the intervention, compostable bins were only in office spaces.^19^

### Stage 3: Intervention evaluation

#### Design

**Figure 3.**
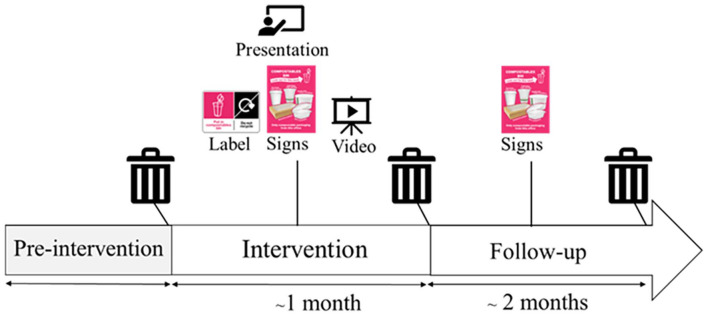
Overview of study procedure. 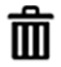
 = indicates when waste assessments were conducted. 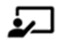
 = presentation from behavioural scientists to workplace leads. 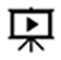
 = motivational video was displayed in workplace canteens and distributed via email/internal communication channels.

A pre–post design was used to assess the effect of the intervention on the percentage of waste in compostable bins that was compostable packaging versus other materials, that is, contamination (primary outcome) and on perceived capability, opportunity and motivation to appropriately dispose of compostable packaging (secondary outcome). During the pre-intervention phase, waste audits were conducted to assess baseline levels of compostable packaging collected and rates of contamination. The intervention phase lasted for 1 month (see Figure 3). Prior to the start of the intervention, contractors applied labels to packaging at each workplace. Workplaces were provided with further labels to apply if the supply ended before the post-intervention waste audit.^20^ Workplace leads briefed catering staff to ensure they used the labelled packaging. Hubbub installed signage on the bins in workplace canteens and office areas. A4-sized posters were displayed by compostable bins on office floors. A6-sized signs were used near cutlery in the canteen. Workplace leads were provided with a link to the motivational video to display on screens in canteens, to email to staff and/or circulate via internal communication channels. The on-boarding presentation was delivered live online to workplace leads within the first 2 weeks of the intervention and a pre-recorded version was shared. If workplace leads were unable to attend a live presentation, a pre-recorded presentation was provided.

The follow-up phase started after post-intervention measures and lasted for 1 month. During this time, signage remained in place, and no notification was provided to workplaces to stop playing the video. Follow-up waste audits were conducted 2 months after the intervention had started. Waste was also assessed at a workplace not participating in the intervention to provide a comparison to the workplaces where the intervention was delivered (see Supplemental Material S5).

Online surveys assessing perceived capability, opportunity and motivation to compost packaging were circulated to workplaces at pre-intervention and post-intervention (see https://osf.io/qfkvc). Based on a medium effect size (*f* = 0.15; Allison et al., 2022a), power calculations in G*Power with an α of 0.05 and power between 0.80 and 0.95 indicated that 78–120 survey participants would be sufficient to detect changes between pre-, post-intervention and follow-up.

### Measures

#### Waste measures

Assessment of the material in the compostables, DMR and general waste bins at workplaces was conducted by an auditor.^21^ Three planned audits were conducted (pre-, post-intervention and follow-up) each on 1-weeks’ collected waste. Further (unplanned) audits were conducted where possible. Each audit followed standardized procedures and randomly selected and weighed waste sacks from bins. The waste was then segregated into different types of waste (e.g. compostable packaging, food, recyclables) and weighed, allowing the percentage of each type of waste in each waste stream to be computed. The primary measures were the percentage (and weight in kilograms) of waste in compostable bins that was (i) compostable packaging and (ii) contaminating materials (not including food^22^). Based on industry insights informed by project partners, a 10% change in the amount of compostable packaging or contaminating materials in compostable bins was considered a meaningful change.

The percentage of waste that was compostable packaging in the DMR, and general waste bin was recorded as secondary waste measures, to identify whether there were changes in the amount of compostable packaging contaminating other waste streams. The pre-, post-intervention and follow-up waste audits were completed at all workplaces. For the duration between the start of the intervention and post-intervention and follow-up waste audits, see Supplemental Material S6.

### Survey

#### Capability, opportunity and motivation

Survey items were developed and rated on a 5-point scale to assess perceived capability, opportunity and motivation to identify and appropriately dispose of compostable packaging (see Supplemental Table S7). Higher scores indicated greater perceived capability, opportunity and/or motivation. Participants also reported demographics and canteen use. The post-intervention survey included two attention-check questions, and participants incorrectly answering both were excluded. The post-intervention survey asked participants if: (i) they had noticed each intervention component (i.e. packaging labels, bin signage and video; yes, no, unsure) and (ii) which component(s) they believed were most effective in helping them to put compostable packaging into compostable bins (see Supplemental Material S8).

#### Intervention acceptability and self-efficacy

The acceptability of the intervention was assessed in the post-intervention survey using a modified version of the Theoretical Framework of Acceptability (Sekhon et al., 2017, 2018), which assessed affective attitude, burden, perceived effectiveness, intervention coherence, self-efficacy, opportunity costs and general acceptability of the intervention^23^ (see https://osf.io/36pq2). Higher scores indicated greater agreement with each item. Self-efficacy to compost packaging at work was measured at pre- and post-intervention to assess whether self-efficacy changed in response to the intervention.

After the follow-up phase, a roundtable event was held with workplace leads and partner organizations to assess workplace leads’ experiences and acceptability of the intervention. Workplace leads completed an online survey assessing the acceptability of the intervention (Sekhon et al., 2018). The perceived usefulness of each intervention component and likelihood that workplaces would continue using compostable packaging were assessed using a 5-point scale (higher scores indicated greater usefulness and continued use). An audio-recorded discussion (transcribed verbatim) then took place, which asked workplace leads what they thought worked well, points for improvement, responses to the proposed recommendations and whether they believed the trial had impacted wider waste management practices at their workplace.^24^

#### Intervention fidelity

Workplace visits were conducted to assess whether the intervention components had been delivered as intended towards the end of the intervention phase.^25^ Intervention components were rated on a 3-point scale (0 = low, 1 = medium and 2 = high intervention fidelity; for criteria and procedure see https://osf.io/csjpe^26^). The evidence obtained suggested that the interventions were delivered as intended (see Supplemental Material S9).

## Results

### Waste measures

**Figure 4.**
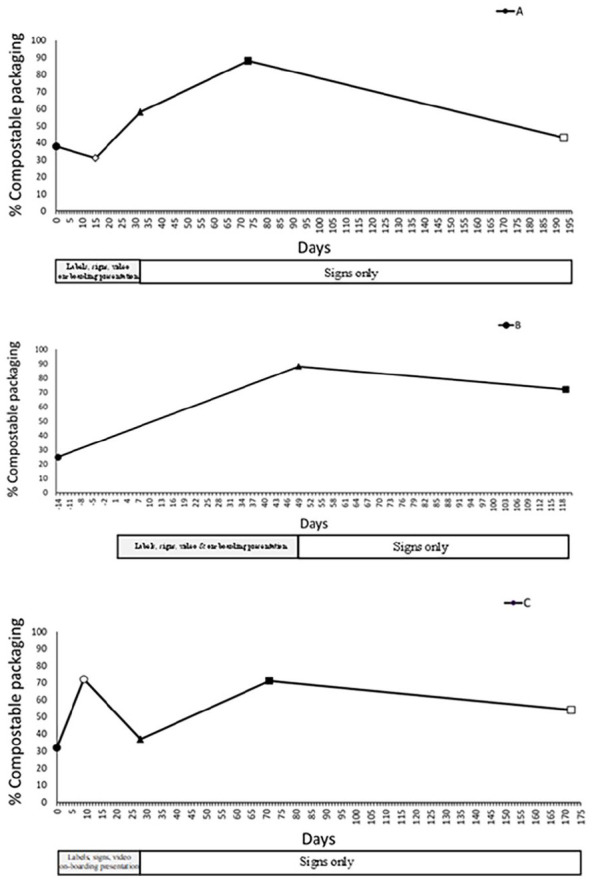
Percentage of waste in the compostable bins that was compostable plastic packaging at pre-, post-intervention, follow-up and ~ 6 months at workplaces A, B and C. Marker shapes indicate waste assessments at pre-intervention (•); ~2 weeks after the intervention started (ο); post-intervention (▲), follow-up (■) and~6 months (□). For days, the intervention started on day 1. Minus numbers indicate the number days between the pre-intervention waste assessment and intervention start date. Workplaces A and C had additional waste assessments 10 and 15 days after the intervention started (ο) and at ~6 months (□). No waste assessments were conducted at workplace B at these time points (see Supplemental Material S6 for details).

Before the intervention, the percentage of waste that was compostable packaging in compostable bins was 38% (workplace A), 25% (workplace B) and 32% (workplace C). This increased between pre- and post-intervention by 20% (workplace A), 63% (workplace B) and 5% (workplace C; for percentages and weights, see Supplemental Table S10 and Figure 4). The amount of compostable packaging collected from compostable bins was higher at follow-up compared to pre-intervention with increases of 50% (workplace A), 47% (workplace B) and 39% (workplace C). Averages across the three workplaces showed increases in the percentage of waste that was compostable packaging between pre-intervention (32%), post-intervention (61%) and follow-up (77%). Changes in the weight (kilograms) of compostable packaging in compostable bins corresponded with the changes in the percentage of waste that was compostable in the bins, suggesting that there was not simply less in the bins in general (Supplemental Table S10).^27^

The increased amount of compostable packaging in compostable bins was mirrored by a reduction in contaminating materials in bins between pre-intervention, post-intervention and follow-up at workplaces A and B (see Supplemental Table S11). Not including food waste, contamination decreased between pre-intervention and post-intervention by 21% (workplace A) and 61% (workplace B) and remained lower at follow-up [reductions of 39% (workplace A) and 57% (workplace B) between pre-intervention and follow-up]. Workplace C’s contamination levels were low at pre-intervention (8%), and contamination increased by 10% at post-intervention, due to increased general waste deposited in compostable bins. Contamination reduced to zero at follow-up.

At workplaces A and B, the percentage of waste in the DMR that was compostable packaging reduced by 10% and 9%, respectively, between pre-intervention and post-intervention (see Supplemental Table S10). This change was not sustained at follow-up. For workplace C, there was a low amount of compostable packaging in the DMR bins at pre-intervention, and this remained throughout the trial. For the general waste bin, there were low levels of compostable packaging at workplace A at pre-intervention. This remained low at post-intervention but increased by 10% at follow-up. For workplace B at post-intervention, there were slight reductions in the percentage of waste that was compostable packaging which was sustained at follow-up. At workplace C, there were small increases in the percentage of waste that was compostable packaging at post-intervention (4%), which returned to a small percentage at follow-up. For a summary of food waste in compostable bins, see Supplemental Material S12.

### Additional unplanned waste assessments

During the trial, opportunity arose for further waste assessments of compostable bins at workplaces A and C, 2 weeks and ~6 months after the intervention started. No additional waste assessments were conducted at workplace B (see Supplemental Material S6 for details). Between pre-intervention and ~2 weeks, at workplace A, there was a 7% decrease, and workplace C a 40% increase in the percentage of waste that was compostable packaging. Six months after the intervention started, both workplaces showed reductions compared to follow-up in the amount of waste in compostable bins that was compostable packaging. For workplace A, this reduced to 43%, which was similar to pre-intervention. At workplace C, the amount reduced to 54% which remained 22% greater than at pre-intervention (see Supplemental Table S10).

### Survey responses

Eighty-five participants at pre-intervention and ninety-seven at post-intervention completed all questions assessing aspects of the COM-B model.^28^ Supplemental Table S13 shows participant characteristics. Beliefs about psychological capability, physical opportunity, social opportunity, automatic motivation and reflective motivation did not significantly differ between pre- and post-intervention (*F*(5, 176) = 1.99, *p* = 0.08, η*p*² = 0.05; see Table 1).

**Table 1.** Capability, Opportunity, Motivation-Behavior (COM-B) components pre- and post-intervention (pre: *n* = 85; post *n* = 97).

COM-B component	Mean SD	
	Pre-intervention	Post-intervention
Psychological capability	3.82 ± 0.64	3.68 ± 0.73
Physical opportunity	3.52 ± 0.76	3.64 ± 0.82
Social opportunity	3.10 ± 0.84	3.21 ± 0.88
Reflective motivation	4.38 ± 0.47	4.32 ± 0.46
Automatic motivation	4.12 ± 0.75	3.88 ± 0.87

Possible scores ranged between 1 and 5 with higher scores indicating greater perceived capability, opportunity and motivation.

SD: standard deviation.

### Intervention acceptability

Mean acceptability scores for each of the TFA constructs indicated good intervention acceptability across all domains (see Supplemental Table S14). Average levels of self-efficacy to compost at work did not significantly change between pre- and post-intervention (*t*(173) = 0.86, *p* = 0.65).

Survey responses collected during the roundtable event are in Supplemental Table S15 (workplace leads *n* = 9).^29^ All intervention components were rated as useful or extremely useful by at least seven leads. Workplace Leads’ acceptability scores showed good levels of intervention acceptability across most domains. Main themes identified from the roundtable discussions with workplaces are in Supplemental Material S16.

## Discussion

Findings suggest that a theory-informed intervention was effective at increasing the percentage of waste in compostable bins that was compostable packaging between pre- and post-intervention. Such improvements were sustained and increased at follow-up and were mirrored by reductions in contaminating materials in compostable bins. However, there were no significant changes in perceived capability, opportunity and motivation between pre- and post-intervention.

Increases in the proportion of compostable packaging in compost bins surpassed the industry-relevant threshold of 10% between pre- to post-intervention at two workplaces. All three workplaces demonstrated meaningful improvements at follow-up (39%, 47% and 50%). The consistency and size of these effects suggest that the interventions changed employees’ behaviour and improved the disposal of compostable packaging. However, there were some inconsistencies. At post-intervention, there was only a 5% increase in compostable packaging in compostable bins at workplace C. This relatively small change did, however, stand in contrast to the 40% increase reported at 2 weeks after the intervention started and the 40% increase observed at follow-up. Workplace A had a 7% reduction in compostable packaging in compostable bins 2 weeks after the intervention started, which differed to the 20% and 50% increases reported at post-intervention and follow-up, respectively. Discussions with project partners suggested that these inconsistencies were likely attributable to isolated contamination incidents. The nature of the waste collection process means that a small number of outlying incidents can impact the quality of the compostable material collected. As such, while there was evidence that the intervention was generally effective, additional steps may be needed to reduce outlying incidents. Furthermore, waste audits at ~6 months showed that the amount of compostable packaging in compostable bins had declined at two workplaces, with one workplace returning to similar amounts to that observed before the intervention. As such, while the intervention was found to have positive effects at ~2-months follow-up, these effects were not necessarily sustained in the longer term.

Nevertheless, the short-term effectiveness of the intervention, aligns with a meta-analysis which reported medium-to-large effects of interventions designed to reduce plastic waste (Allison et al., 2022a) and extends such findings to the disposal of compostable packaging. The main intervention functions included environmental restructuring and persuasion (distinctive labels and signs, video) which have been previously found to be effective for promoting sustainable waste behaviours (Allison et al., 2022b). These findings are important as, while compostable packaging can offer a more sustainable alternative to some conventional plastics, the environmental benefits depend on the appropriate disposal of compostable packaging so that it can be industrially composted and not contaminate other waste streams.

The study did not find significant changes in perceived capability, opportunity and motivation to appropriately dispose of compostable packaging in response to the intervention. One potential explanation is that the intervention prompted employees to appropriately dispose of compostable packaging without much conscious engagement with the intervention components and thus changes in employees’ beliefs about their capability, opportunity and motivation. Indeed, some of the intervention components served as cues to action (e.g. labels on packaging and on bins) and so may have triggered the desired behaviours without the need for conscious deliberation. Additionally, once these cues were removed at ~6 months, the effects of the intervention reduced at two workplaces. We therefore extend calls for greater consideration of nonconscious processes in health (Sheeran et al., 2013) to research on sustainable action. Of note, changes in behaviour without corresponding changes in perceived capability, opportunity and motivation is inconsistent with the COM-B model (which would predict that changes in behaviour follow from changes in perceived capability, opportunity and/or motivation). Our findings suggest that the COM-B model may be more suited to identifying barriers and enablers and designing intervention components than evaluating the effects of interventions. Indeed, to date, most research has applied the COM-B model to identify barriers and enablers of a targeted behaviour and inform the design of an intervention, and not subsequently evaluated the intervention (e.g. Allison et al., 2022b). However, further research, similar to the present study which uses the COM-B model to design *and* evaluate the effects of an intervention, is needed to confirm this.

The COM-B model (Michie et al., 2011) has mostly been used to encourage health behaviours (Michie et al., 2011). However, the present study adds to emerging studies applying the COM-B model to encourage pro-environmental behaviours such as gardening in front gardens (Allison et al., 2024) repairing and repurposing clothing (Zhang and Hale, 2022), and shifting to plant-based diets (Kuosmanen et al., 2024). The behavioural changes reported in the present study support future application of the COM-B model to inform the design of interventions to promote pro-environmental behaviours.

The practical implications of the research are clear – it provides an intervention that could accompany the implementation of new and/or improvement of existing composting schemes. Having consistent, clear and distinctive bin signage that corresponds with standardized packaging labels (colour-coded) and motivational messages can support the appropriate disposal of compostable packaging. To support effective implementation of consistent and clear bins and signage, it is recommended that a credible source communicates with organizations to highlight the importance of delivering evidence-based communications in a consistent way. Organizations delivering composting schemes should also provide bins for compostable material in locations where compostable packaging is disposed of. Furthermore, staff need to be regularly encouraged in waste initiatives to minimize outlying incidents which impact the quality of waste collected. These recommendations are relevant to compostable packaging producers (provide compostable bins and distinctive and clear bin signage to clients, along with training from a credible source) and workplaces implementing composting schemes within closed loop contexts (display signage and communications provided by compostable packaging producers, engage in training from credible sources).

The research has wider policy-related implications. There is no standardized labelling or packaging requirements in the United Kingdom for compostable packaging. There have been calls for standardized distinctive markings for compostable packaging (Allison et al., 2022b; Closed Loop Partners’ Composting Consortium & Biodegradable Products Institute, 2023; WRAP, 2020). However, current legislation is limited and the compostable packaging sector has been referred to as a ‘Wild West’ given the range of brands producing compostable packaging and limited regulations for this packaging (House of Commons, 2022). There is an urgent need for a standardized and regulated label that can be used on compostable packaging.

Strengths of this work include the use of objective waste assessments and evaluating a systematically developed, theory-based intervention in real-world contexts. However, in this study, it was not possible to randomize workplaces to conditions (Craig et al., 2008). The pre–post design meant it was not possible to isolate and test the effects of each specific intervention component, nor compare the intervention to a control. The relatively small number of workplaces who took part may also reflect those who are most engaged in sustainability. However, rates of composting were relatively low at all three workplaces and therefore, the sample represented workplaces where improvements to composting were needed.

## Conclusion

The present research used an approach described by the BCW to develop and evaluate the effects of an intervention designed to promote the appropriate disposal of compostable packaging in workplace canteens. There was evidence that the intervention increased the amount of compostable packaging in the relevant bins and reduced contamination. Based on these findings, it is recommended that closed loop systems use consistent, clear and distinctive bin signage which visually corresponds to standardized packaging labels (e.g. colour-matching) to prompt automatic identification of compostable packaging and appropriate disposal behaviours. It is also recommended that workplaces deliver communications related to compostable packaging in a consistent way and regularly engage and involve staff, highlighting the relatable benefits of compostable packaging.

## Supplemental Material

sj-docx-1-wmr-10.1177_0734242X251322145 – Supplemental material for Developing and evaluating an intervention to improve the disposal of compostable packaging at UK workplaces Supplemental material, sj-docx-1-wmr-10.1177_0734242X251322145 for Developing and evaluating an intervention to improve the disposal of compostable packaging at UK workplaces by Nicola J Buckland, Sara Bru Garcia, Rosie Sharp, Tom Mockridge, Sarah Greenwood, Meghann Matthews and Thomas L Webb in Waste Management & Research

## Notes

1. In other words, an individual needs to be capable, motivated and have the opportunity to put compostable packaging in a bin.

2. Closed loop contexts also allow for compostable packaging to be collected and processed with food scraps, which can divert food waste away from landfill (Song et al., 2009).

3. Stage 1 also involved regular meetings between partners.

4. All three workplaces that completed the intervention had participated in the observations and focus groups.

5. Deviations from the pre-registered protocols are reported where relevant.

6. The Behaviour Change Technique Ontology (Marques et al., 2023) became available after the intervention development process; therefore, references to the Behaviour Change Taxonomy v1 are primarily reported.

7. Due to commercial sensitivity, the precise content of the materials cannot be shared.

8. To participate, participants had to be ⩾18 years old and report using the canteen at least once a week or be kitchen/cleaning staff. Participants were offered a £10 shopping voucher. The number of focus groups was less than the targeted sample size pre-registered (three groups per workplace); however, the researchers reported no new themes being identified within the last two groups and the sample size was at the upper end of recommendations for meeting data saturation (Hennink and Kaiser, 2022).

9. Themes were mapped to COM-B through group discussion (NJB, SBG, TLW). The pre-registration protocol planned for two researchers to independently map themes to COM-B components. However, at the point of mapping, group discussions were deemed more conducive.

10. The process for observations were not pre-registered as this was led by a non-academic partner.

11. For example, at one workplace, there were five types of coffee cups, two were compostable and three were recyclable. Yet, there was limited visual distinction (e.g. no clear labels) between the compostable and recyclable packaging and bins.

12. The other three workplaces had compostable bins in the canteen.

13. Using an online whiteboard (Miro).

14. The selection of intervention components was based on potential effectiveness and feasibility to implement across workplaces, along with the potential for wider upscaling which was informed through discussions with partners.

15. A not-for-profit company who provide a certified recyclable label to UK businesses.

16. Market research with 200 members of a research panel who ranked label preferences based on how understandable and eye-catching each label was.

17. Stickers were made of compostable materials.

18. The video used in the intervention had pink framing and showed the packaging label developed and used in the trial. These aspects have been adapted in the version shared here. However, the scenes and main messages are unchanged.

19. This intervention component arose via workplace A’s request (not via the formal intervention development process).

20. Workplaces A and C applied further stickers themselves. For workplace B, temporary contractors applied further stickers.

21. The auditor was blind (unaware) to the aims of the research.

22. Food was not considered a contaminant because it degrades as part of the composting process and use of compostable packaging can beneficially re-direct food waste from landfill to composting.

23. The theoretical framework of acceptability also assessed ethical consequences, which was not assessed in this study due to an error whereby the relevant question was not included in the survey.

24. Project partners also discussed the intervention in separate discussions to the workplaces, which is not reported here.

25. Deviation from the pre-registered protocol – fidelity checks were planned to be conducted once during the intervention and once during the follow-up phase.

26. The 3-point scale deviated from the pre-registered 6-point scale for ease of use and the specific criteria for each score slightly varied to the pre-registered protocol to align more with each intervention component.

27. Average weights across the three workplaces also showed an increase between pre-intervention (5.4 kg), post-intervention (10.4 kg) and follow-up (12.21 kg).

28. For the pre-intervention survey, there were 102 initial responses, of which 94 participants consented to take part. Eighty-five and eighty-one continued to complete the COM-B and demographic questions, respectively. For the post-intervention survey, there were 134 initial responses, of which 114 provided consent. Three responses were removed due to incorrect responses to attention check questions (*n* = 2) or requesting withdrawal (*n* = 1). Ninety-seven and ninety-three continued to complete all COM-B and acceptability/demographic questions, respectively. Participants were retained in the analysis up to the point of dropping out.

29. Workplace A, n = 3; workplace B, n = 2; workplace C, n = 4.
