# Supplementary material for: Developing and evaluating an intervention to improve the disposal of compostable packaging at UK workplaces
Source: Waste Manag Res. 2025 Feb 26;43(10):1625–35. doi: 10.1177/0734242X251322145 (PMC12476459; doi:10.1177/0734242X251322145)
Supplement: sj-docx-1-wmr-10.1177_0734242X251322145 – Supplemental material for Developing and evaluating an intervention to improve the disposal of compostable packaging at UK workplaces [file sj-docx-1-wmr-10.1177_0734242X251322145.docx]

**Developing and evaluating an intervention to improve the disposal of compostable packaging at UK workplaces: SUPPLEMENTARY MATERIALS**

Nicola J Buckland^a^, Sara Bru Garcia^a^, Rosie Sharp^b^, Tom Mockridge^c^, Sarah Greenwood^d,e^, Meghann Matthews^a^, Thomas L Webb^a^

^a^School of Psychology, University of Sheffield, ICOSS Building, 219 Portobello, Sheffield, S1 4DP.

^b^Hubbub**,** Somerset House, Strand, London, WC2R 1LA.

^c^Recorra, 52 Lant Street, London SE1 1RB.

^d^Department of Chemistry, University of Sheffield, Sheffield S3 7HF.

^e^Grantham Centre for Sustainable Futures, University of Sheffield, Sheffield S3 7RD.

**Corresponding author:** Dr Nicola Buckland: [n.buckland@sheffield.ac.uk](mailto:n.buckland@sheffield.ac.uk)

### **List of Supplementary Materials**

### S1 Table. A description of the existing materials.

### S2 Table. Coding existing materials offered to workplaces.

### S3 Table. Thematic codebook generated from focus groups.

### S4 Table. Intervention components mapped to COM-B components.

### S5 Text. Waste assessments at a workplace not participating in the intervention.

### S6 Text. Duration between pre-intervention, post-intervention and follow-up.

### S7 Table. Survey items assessing each COM-B component.

### S8 Text. Awareness of intervention components.

### S9 Text. Assessing intervention fidelity.

S10 Table. Percentage and weight (kg) of waste in the compostables, dry mixed recycling and general waste bins at pre-intervention, post-intervention and follow-up.

S11 Table. Contamination and food in the compostable bins.

S12 Text. Summary of food waste in compostable bins.

S13 Table. Characteristics of survey participants who completed the pre-intervention and post-intervention surveys.

S14 Table. Acceptability of the intervention for employees and workplace leads.

S15 Table. Roundtable survey responses.

### S16 Table. Workplace Leads’ ratings of the intervention during the roundtable event**.**

**Supplementary Materials 1, Table (S1). A description of the existing materials.**

| **Item** | **Description** |
| --- | --- |
| A | Training manual for workplaces |
| B | Fact sheet with steps to implementing and maintaining a compost scheme |
| C | Packaging/container product list |
| D | Bin signage template |
| E | Video on compostable packaging and the composting process |
| F | Signs on tables about compostable packaging |
| G | Poster for canteens showing items that can be composted |
| H | Poster for canteens showing composting process |
| I | Infographic on composting process |
| J | Poster encouraging customers to return compostable packaging for composting |
| K | Sticker showing a compostable cup to display on fridges or windows |
| L | Sign at tills showing a compostable bin and instructions to compost |
| M | Training document for cleaning/catering staff |
| N | Training presentation for cleaning/catering staff |
| O | Kick start training event for staff |
| Pi | Case study on a University workplace with an effective composting scheme (PDF, blog, video and LinkedIn post) |
| Pii | Case study on a Science Centre workplace with an effective composting scheme (PDF, blog and video) |
| Piii | Case study on a University workplace (different University to Pi) with an effective composting scheme (PDF, blog and video) |

**Supplementary Materials 2, Table (S2)**. **Coding intervention functions, COM-B components, TDF domains, the presence of behaviour change techniques (BCTs) and modes of delivery in existing materials offered to workplaces.**

|  | **A** | **B** | **C** | **D** | **E** | **F** | **G** | **H** | **I** | **J** | **K** | **L** | **M** | **N** | **O** | **Pi** | **Pii** | **Piii** | **Total** |
| --- | --- | --- | --- | --- | --- | --- | --- | --- | --- | --- | --- | --- | --- | --- | --- | --- | --- | --- | --- |
| **Intervention functions** |  |  |  |  |  |  |  |  |  |  |  |  |  |  |  |  |  |  |  |
| Education | ✓ | ✓ | ✓ | ✓ | ✓ | ✓ | ✓ | ✓ | ✓ | ✓ | ✓ | ✓ | ✓ | ✓ | ✓ | ✓ | ✓ | ✓ | **18** |
| Persuasion | ✓ | ✓ | ✓ | - | ✓ | ✓ | ✓ | ✓ | ✓ | ✓ | - | ✓ | - | - | ✓ | ✓ | ✓ | ✓ | **14** |
| Incentivisation | ✓ | ✓ | - | - | - | - | - | - | - | - | - | - | - | - | ✓ | - | - | - | **3** |
| Coercion | - | - | - | - | - | - | - | - | - | - | - | - | - | - | - | - | - | - | **0** |
| Training | ✓ | - | - | - | - | - | - | - | - | - | - | - | ✓ | ✓ | ✓ | - | - | - | **4** |
| Restriction | - | - | - | - | - | - | - | - | - | - | - | - | - | - | - | - | - | ✓ | **1** |
| Environmental restructuring | ✓ | - | ✓ | ✓ | - | - | - | - | - | - | - | - | - | - | ✓ | - | - | - | **4** |
| Modelling | - | - | - | - | - | - | - | - | - | - | - | - | - | - | - | ✓ | ✓ | ✓ | **3** |
| Enablement | ✓ | ✓ | ✓ | - | - | - | - | - | - | ✓ | - | - | - | - | ✓ | ✓ | - | - | **6** |
|  |  |  |  |  |  |  |  |  |  |  |  |  |  |  |  |  |  |  |  |
| **COM-B components** |  |  |  |  |  |  |  |  |  |  |  |  |  |  |  |  |  |  |  |
| Physical capability | - | - | - | - | - | - | - | - | - | - | - | - | - | - | ✓ | - | - | - | **1** |
| Psychological capability | ✓ | ✓ | ✓ | ✓ | ✓ | ✓ | ✓ | ✓ | ✓ | ✓ | ✓ | ✓ | ✓ | ✓ | ✓ | ✓ | ✓ | ✓ | **18** |
| Physical opportunity | ✓ | ✓ | ✓ | ✓^a^ | ✓^a^ | ✓^a^ | ✓ | ✓^a^ | ✓^a^ | ✓ | ✓ | ✓ | - | - | ✓ | - | ✓ | - | **14** |
| Social opportunity | ✓ | - | - | - | - | - | - | - | - | - | - | - | - | - | ✓ | ✓ | ✓ | ✓ | **5** |
| Reflective motivation | ✓ | ✓ | ✓ | - | ✓ | ✓ | ✓ | ✓ | ✓ | ✓ | - | - | - | - | - | ✓ | ✓ | ✓ | **12** |
| Automatic motivation | ✓ | - | - | - | - | - | - | - | - | - | - | - | - | - | ✓ | ✓ | ✓ | - | **4** |
|  |  |  |  |  |  |  |  |  |  |  |  |  |  |  |  |  |  |  |  |
| **Theoretical Domains Framework** |  |  |  |  |  |  |  |  |  |  |  |  |  |  |  |  |  |  |  |
| Knowledge | ✓ | ✓ | ✓ | ✓ | ✓ | ✓ | ✓ | ✓ | ✓ | ✓ | ✓ | ✓ | ✓ | ✓ | ✓ | ✓ | ✓ | ✓ | **18** |
| Skills | - | - | - | - | - | - | - | - | - | - | - | - | - | - | ✓ | - | - | - | **1** |
| Memory, attention and decision processes | - | - | - | ✓ | - | - | - | - | - | - | - | - | - | - | ✓ | - | - | - | **2** |
| Behavioural regulation | - | - | - | - | - | - | - | - | - | - | - | - | - | - | - | - | - | - | **0** |
| Social/professional role and identity | ✓ | - | - | - | - | - | - | - | - | - | - | - | ✓ | - | - | - | - | - | **2** |
| Beliefs about capabilities | - | ✓ | - | - | - | - | - | - | - | ✓ | - | - | - | - | ✓ | ✓ | - | ✓ | **5** |
| Optimism | - | - | - | - | - | - | - | - | - | - | - | - | - | - | - | ✓ | - | - | **1** |
| Beliefs about consequences | ✓ | - | ✓ | - | ✓ | - | ✓ | ✓ | ✓ | ✓ | - | - | ✓ | - | - | ✓ | ✓ | ✓ | **11** |
| Intentions | - | - | - | - | - | - | - | - | ✓ | - | - | - | - | - | - | - | - | - | **1** |
| Goals | ✓ | ✓ | - | - | - | - | - | - | - | - | - | - | - | - | - | - | ✓ | - | **3** |
| Reinforcement | ✓ | - | - | - | - | - | - | - | - | - | - | - | - | - | - | ✓ | - | - | **2** |
| Emotion | - | - | - | - | ✓ | - | - | - | - | - | - | - | - | - | - | - | - | - | **1** |
|  |  |  |  |  |  |  |  |  |  |  |  |  |  |  |  |  |  |  |  |
| **Behaviour change techniques** |  |  |  |  |  |  |  |  |  |  |  |  |  |  |  |  |  |  |  |
| 1.1 Goal setting | ✓ | ✓ | - | - | - | - | - | ✓ | ✓ | - | - | - | ✓ | - | ✓ | - | - | - | **6** |
| 1.2 Problem solving | ✓ | - | - | - | - | - | - | - | - | - | - | - | - | - | - | ✓ | ✓ | ✓ | **4** |
| 1.3 Goal setting (outcome) | - | - | - | - | ✓ | - | - | - | - | - | - | - | - | - | - | - | - | ✓ | **2** |
| 1.4 Action Planning | - | - | - | - | - | - | - | - | - | - | - | - | - | - | ✓ | - | - | - | **1** |
| 2.1 Monitoring of behavior by others without feedback | ✓ | - | - | - | - | - | - | - | - | - | - | - | - | - | - | - | - | - | **1** |
| 2.2 Feedback on behaviour | - | - | - | - | - | - | - | - | - | - | - | - | - | - | - | - | - | ✓ | **1** |
| 2.4 Self-monitoring of outcome(s) of behavior | ✓ | - | - | - | - | - | - | - | - | - | - | - | - | - | - | - | - | - | **1** |
| 2.5 Monitoring of outcome(s) of behavior by others without feedback | - | - | - | - | - | - | - | - | - | - | - | - | ✓ | - | - | - | - | - | **1** |
| 2.7 Feedback on outcome(s)  of behavior | ✓ | - | - | - | - | - | - | - | - | - | - | - | - | - | - | - | - | - | **1** |
| 3.1 Social support (unspecified) | ✓ | ✓ | ✓ | - | - | - | - | - | - | - | - | - | ✓ | ✓ | ✓ | ✓ | - | - | **7** |
| 3.2 Social support (practical) | ✓ | ✓ | ✓ | - | - | - | ✓ | - | - | ✓ | - | ✓ | - | - | ✓ | ✓ | ✓ | - | **9** |
| 4.1 Instruction on how to perform a behavior | ✓ | ✓ | - | ✓ | - | - | ✓ | ✓ | - | ✓ | - | ✓ | ✓ | ✓ | ✓ | ✓ | ✓ | ✓ | **13** |
| 4.2 Materials on antecedents | - | - | - | - | - | - | - | - | - | - | - | - | - | - | - | ✓ | - | ✓ | **3** |
| 5.2 Salience of consequences | - | - | - | - | ✓ |  | - | - | ✓ | ✓ | - | - | ✓ | - | - | ✓ | - | - | **4** |
| 5.3 Materials about social and environmental consequences | ✓ | ✓ | ✓ | - | ✓ | ✓ | ✓ | ✓ | ✓ | ✓ | - | - | - | ✓ | ✓ | ✓ | ✓ | ✓ | **14** |
| 6.1 Demonstration of the behaviour | - | - | - | - | - | - | - | - | - | - | - | - | - | - | - | - | - | ✓ | **1** |
| 6.2 Social comparison | - | - | - | - | - | - | - | - | - | - | - | - | - | - | ✓ | - | - | - | **1** |
| 6.3 Materials about others’ approval | - | - | - | - | - | - | - | - | - | - | - | - | - | - | - | ✓ | - | ✓ | **2** |
| 7.1 Prompts / cues | - | - | - | ✓ | - | ✓ | - | - | - | - | ✓ | ✓^a^ | - | - | ✓ | - | - | ✓ | **5** |
| 8.1 Behavioural practice / rehearsal | - | - | - | - | - | - | - | - | - | - | - | - | - | - | ✓ | - | - | - | **1** |
| 8.2 Behavior substitution | ✓ | - | - | - | - | - | - | - | - | - | - | - | - | - | - | - | - | ✓ | **2** |
| 8.6 Generalisation of target behaviour | - | - | - | - | - | - | - | - | - | - | - | - | - | - | - | - | - | ✓ | **1** |
| 8.7 Graded tasks | ✓ | - | - | - | - | - | - | - | - | - | - | - | - | - | - | - | - | ✓ | **2** |
| 9.1 Credible source | ✓ | ✓ | ✓ | - | - | - | - | - | - | - | - | - | - | - | ✓ | ✓ | ✓ | ✓ | **7** |
| 10.2 Material reward (behaviour) | - | - | - | - | - | - | - | - | - | - | - | - | - | - | ✓ | - | - | - | **1** |
| 10.3 Non-specific reward | - | - | - | - | - | - | - | - | - | - | - | - | - | - | ✓ | - | - | - | **1** |
| 10.4 Social reward | ✓ | - | - | - | - | - | - | - | - | - | - | - | - | - | - | - | - | ✓ | **2** |
| 12.1 Restructuring the physical environment | ✓ | - | - | - | - | - | - | - | - | - | - | - | - | - | - | - | - | - | **1** |
| 12.5 Adding objects to the environment | - | - | ✓ | - | - | - | - | - | - | - | - | - | - | - | ✓ | ✓ | - | - | **3** |
| 13.1 Identification of self as role model | ✓ | - | - | - | - | - | - | - | - | - | - | - | - | - | ✓ | - | - | - | **2** |
| 15.1 Verbal persuasion about capability | - | ✓ | - | - | - | - | - | - | - | - | - | - | - | - | - | ✓ | - | ✓ | **3** |
| 16.3 Vicarious consequences | - | - | - | - | - | - | - | - | - | - | - | - | - | - | - | ✓ | - | - | **1** |
| **Total BCTs** | **15** | **7** | **5** | **2** | **3** | **2** | **3** | **3** | **2** | **4** | **1** | **3** | **5** | **2** | **14** | **12** | **5** | **15** |  |
|  |  |  |  |  |  |  |  |  |  |  |  |  |  |  |  |  |  |  |  |
| **Mode of delivery^b^** |  |  |  |  |  |  |  |  |  |  |  |  |  |  |  |  |  |  |  |
| Human interactional; BCIO:011002 | **-** | **-** | **-** | **-** | **-** | **-** | **-** | **-** | **-** | **-** | **-** | **-** | **-** | ✓ | **-** | **-** | **-** | **-** | **1** |
| Electronic billboard; BCIO:011014 | **-** | - | **-** | - | ✓ | - | - | - | - | - | - | - | - | - | - | - | - | - | **1** |
| Public notice; BCIO:011007 | **-** | - | **-** | ✓ | **-** | ✓ | ✓ | ✓ | ✓ | ✓ | ✓ | ✓ | **-** | **-** | **-** | **-** | **-** | **-** | **8** |
| Printed publication; BCIO:011008 | ✓ | ✓ | ✓ | **-** | **-** | **-** | **-** | **-** | **-** | **-** | **-** | **-** | ✓ | **-** | ✓ | ✓ | ✓ | ✓ | **8** |
| Electronic; BCIO:011010 | **-** | **-** | **-** | **-** | **-** | **-** | **-** | **-** | **-** | **-** | **-** | **-** | **-** | **-** | **-** | ✓ | ✓ | ✓ | **3** |
| Computer; BCIO:011013 | **-** | **-** | **-** | **-** | **-** | **-** | **-** | **-** | **-** | **-** | **-** | **-** | **-** | ✓ | **-** | **-** | **-** | **-** | **1** |

*Note.*

The process for coding involved two coders independently coded two initial materials and comparing coding. Discrepancies were discussed and then both coders independently coded the remaining materials. The coding was compared between coders and any discrepancies were discussed and the final coding agreed. There were no major disagreements between the coders.

^a^BCT was not initially coded, however when results were tabulated the coding was updated in a final review process to confirm all materials had been coded consistently.

^b^The most specific level for mode of delivery was coded.

COM-B = capability, opportunity, motivation-behaviour model.

TDF = Theoretic Domains Framework.

The process for coding involved two coders independently coded two initial materials and comparing coding. Discrepancies were discussed and then both coders independently coded the remaining materials. The coding was compared between coders and any discrepancies were discussed and the final coding agreed. There were no major disagreements between the coders.

**Supplementary Materials 3, Table (S3). Thematic codebook generated from focus groups.**

| **COM-B Component** | **Theme** | **Barrier/Enabler** | **Example quote** |
| --- | --- | --- | --- |
| Psychological Capability | Awareness about what compostable packaging is | Both | *“Breaks down and can then be reused in some way, shape or form. I don't know if it gets used in traditional compost, where you'd put it on your plants or use it as a soil base, or whether then it turns into some pulp and used in other bits of manufacturing”*  “*Compostable is like something that can easily go into flame, burn, yes, things like that, like plastic cups, cardboard cups, things like that*” |
|  | Knowledge, attention and clarity of packaging, label and brand | Barrier | *“I've been here for 6 years and I've been facilities for 2, I'd see Vegware, I didn't know what Vegware was until someone says, 'This is what Vegware-,' until it's publicised, I don't think it's necessarily clear”*  *“I think there's a logo on the base that says 'Vegware,' but as I say, you've sometimes got to look for it quite hard”*  *“It's definitely the signing on it [...] on the Vegware stuff, they coffee cups are 1 colour, the boxes are another colour. They're embossed, it's not that clear.”* |
|  | Confusion separating waste | Barrier | “*I think there's a lot of confusion out there in terms of what you can actually put in certain waste streams, and I have this dilemma at home, as well”* |
|  | Awareness about the problems with contamination | Barrier | *“And is it, sort of, any recycling that is tainted if it's got food in it? Does it, you know, undo everything that's in there if someone just throws a full coffee cup in there with everything else? Does that, you know, contaminate in any shape or form? I don't know”* |
|  | The difference between recycling and composting | Barrier | *“I wouldn't know if that was a massive error to put it in the recycling bin and not the Vegware bin, or if they are interchangeable”* |
|  | Need for training and education to increase awareness about compostable waste | Barrier | *“I think it's information. They're not informed enough, they don't know. I think it's all about not being informed [...] if they provide, if they educate. So some, kind of a, I don't know. Provide more information”* |
| Social opportunity | social norms around composting | Both | *“I just think in the kitchens, you're more inclined to adhere to it. 1, because 9 times out of 10, there's somebody else around you, and you're more conscious of it”* |
| Physical opportunity | Workplaces are messy contexts | Barrier | *“In the restaurant, they just said, sometimes we have an issue with Vegware orders, we order something else. That other thing, we don't know what it is. I myself, I don't know what it is, so it just goes into general waste, because it can't be recycled”* |
|  | Compostable packaging looks and feels like “normal” packaging | Barrier | *“You could think that that's cardboard, and therefore, the recycling bin. And I don't know if they're interchangeable, but that's an easy mistake to make, because it's quite cardboard-y, the box. It's not like Polystyrene, is it?”* |
|  | Need for clear labelling, prompts and communications on what goes in the compost bin | Barrier | *“It's like, 'It's compostable,' it's not, it's cardboard, so put it in the right-, so just make the bin clearer, the signage easier for them to read and to understand, and to know what Vegware signage as well, could be, 'Compost me,' or something, just, like make, it clearer to them”* |
|  | Bins are sufficient, easy to access and use | Enabler | *“I think they're very easy to access and super easy to use. I mean, they're bright pink, like, you can't miss it.”* |
| Reflective motivation | Composting is good for society and the environment | Enabler | *“Well, helping the environment, that is what we all should be doing, that's the biggest benefit I would say”* |
|  | Beliefs about rewards and punishment as tools to influence behaviour | Barrier | *“The main incentive would be a free treat. People who go, 'Oh, I'll get a free coffee if I do this,' or, 'I get a free meal if I recycle correctly.' Would that happen every day? I don't think so, but then, kind of, a stamp programme, for example”* |
|  | Attitudes towards separating waste and contamination | Both | *“Using takeaway packaging, compostable or non-compostable, it's still bad.”* |
|  | Understanding and relating to the consequences of composting | Barrier | *“If all of a sudden you know what happens to your cup when you put it in that building that it actually produces that, I'm sure that would just be a little lightbulb moment in someone's head, 'Oh actually, yes I should do that more,'”* |
| Automatic motivation | Convenience and work demands take precedence | Barrier | *“I mean, it sounds incredibly lazy you can't be bothered to get up and go to the kitchen but on the whole people don't do it and will just throw it in a general waste at the end of a bank of desks. Or the recycle one”* |
|  | Lack of habit of composting at work | Barrier | *“Because we don't have those bins at home and maybe that's not habit yet. Need time to form the habit”* |

### ***Note.***

Focus groups (1-hour) were conducted in November 2022 and comprised of kitchen, cleaning or office staff from four workplaces (n = 29).

Group sizes ranged between three and seven participants.

Thematic analysis was conducted in NVivo by one researcher (SBG; inductive approach). A second researcher (NJB) reviewed codes and extracts.

**Supplementary Materials 4, Table (S4). Intervention components developed to address barriers related to capability, opportunity and motivation to appropriately dispose of compostable packaging.**

| **Barrier** | **COM-B** | **Intervention** | **Function and BCTs (BCIO #)** | **Mode of delivery and timing of delivery** |
| --- | --- | --- | --- | --- |
| Compostable packaging was difficult to identify and distinguish from other types of packaging (e.g. recyclable packaging).  Confusion separating waste as bins for compostables were not clearly marked and bin signage around which packaging goes in each was unclear.  Exacerbating this barrier, bins and bin signage differed between workplaces. | Psychological capability | 1. Distinctive pink-coloured label with instructions to ‘*Put in compostables bin*’ was applied to all compostable plastic packaging in workplace canteens. 2. Pink coloured signs on and near bins for compostables were provided to prompt people to ‘*Look out for this label*’ and displayed items for the compostable bins.   Colour coding was used for the labels and signage to prompt people to put compostable packaging in the colour coded bin. | Functions: Education;  Environmental restructuring.  BCTs:  7.1 Prompts / cues (BCIO:007081)  4.1 Instruction on how to perform the behaviour (BCIO:007058)  3.2 Social Support (Practical) (BCIO:007040) | 1. Labelling mode of delivery (BCIO:011009) 2. Public notice mode of delivery BCIO:011007   Labels and signs were implemented from Day 1 of the intervention.  Labels were discontinued during the follow-up phase. Signs remained in place throughout the trial. |
| Existing materials provided by Vegware (compostable packaging producer) were not implemented as intended at workplaces.  Insufficient compostable bins in the canteen. | Physical opportunity | 1. On-boarding presentation delivered by behavioural scientists to workplace leads to encourage workplace leads to deliver the intervention as intended. 2. Two bins for compostable packaging were added to workplace A^a^ | Functions: Education  Persuasion  Enablement  Environmental restructuring;  BCTs:  9.1 Credible source (BCIO:007189)  4.1 Instruction on how to perform the behaviour (BCIO:007058)  12.5 Adding objects to the  Environment (BCIO: BCIO:007156) | Video call mode of delivery; BCIO:011023  Environmental change mode of delivery  BCIO:011033  Delivered once during the first two weeks of the intervention. |
| People were unaware or did not relate to the consequences of composting. | Reflective motivation | 1. A video for staff at workplaces highlighted the relatable benefits of composting (Compost feeds fields. Fields feed us). | Functions:  Education  Persuasion  Environmental restructuring  Modelling  BCTs:  5.3 Information about social and environmental consequences (BCIO:007176)  7.1 Prompts / cues (BCIO:007081) | Television mode of  delivery  BCIO:011011  Website mode of delivery  BCIO:011027  Email mode of delivery  BCIO:011025 |

^a^The addition of bins arose from workplace A’s request and plans, not through the formal intervention development processes.

**Supplementary Materials Text, 5 (S5). Waste assessments at a workplace not participating in the intervention.**

Two waste assessments were conducted at another workplace that did not take part in the intervention to provide a descriptive comparison to the workplaces where the intervention was delivered. However, it was not possible to conduct 1- and 2-months waste assessments that corresponded with when the intervention was delivered at workplaces A-C. Therefore, comparisons to the intervention workplaces were not possible. However, for transparency, the two waste assessments are reported. The first waste assessment (conducted within a similar month to the first waste assessments conducted at the intervention workplaces), showed that in the compostable bins, 19% (4.83 kg) of the waste collected was compostable packaging [13% was food waste (3.46 kg)]. The second waste assessment was conducted 230 days later (in the same month that the 6 months waste collections took place at workplaces A and C) and showed 34% (4.38 kg) of the waste collected was compostable packaging [29% was food waste (3.80 kg)].

**Supplementary Materials Text, 6 (S6). Duration between pre-intervention, post-intervention and follow-up.**

For workplaces A and C, waste was assessed at planned time points at post-intervention (workplace A: 32 days; workplace C: 29 days) and follow-up (workplace A: 73; workplace C: 72 days). However, for workplace B, there were delays conducting the post-intervention (64 days) and follow-up audits (119 days) due to logistical challenges accessing the workplace. The compostable bins were also assessed at workplaces A and C at 10 and 15 days after the intervention started (dry mixed recycling and general waste bins were not assessed at these time points). As well as 193 and 174 days after the intervention started (~6 months; A and C respectively). No additional waste audits were conducted for workplace B.

**Supplementary Materials 7, Table (S7). Survey items assessing each COM-B component.**

| **Survey items for each COM-B component** | **Cronbach’s alpha** | |
| --- | --- | --- |
|  | **Pre-** | **Post-** |
| **Psychological capability** | .72 | .74 |
| I am aware that some packaging in the canteen/cafeteria is compostable |  |  |
| I find it difficult to identify if packaging is compostable |  |  |
| I don’t know what to do with compostable packaging after using it |  |  |
| I know what I can and can’t put in the compostables bin at work |  |  |
| I find deciding where to dispose of compostable packaging at work confusing |  |  |
| **Physical opportunity** |  |  |
| The signs and posters on/around the bins clearly explain what waste to put in each bin | .82 | .84 |
| It is clear which bins are for compostable packaging |  |  |
| There are sufficient reminders in the canteen/cafeteria to prompt me to put compostable packaging in the compostables bin |  |  |
| The compostable packaging available at work is clearly labelled as ‘compostable packaging’ |  |  |
| I can easily tell which bin compostable packaging goes in just by looking at the packaging |  |  |
| The layout of the canteen/cafeteria encourages me to put compostable packaging in the compostables bin |  |  |
| **Social opportunity** | .63 | .71 |
| I think that there is a strong culture in my workplace to put compostable packaging in the compostables bin |  |  |
| Most of my colleagues put compostable packaging in the compostables bin |  |  |
| **Reflective motivation** | .65 | .63 |
| Nothing useful is done with compostable packaging that is put in the compostables bin at work |  |  |
| Putting non-compostable packaging in the compostables bin doesn’t cause any issues |  |  |
| It won’t make a difference whether I put compostable packaging in the compostables bin or the general waste bin after using it |  |  |
| It is my responsibility to put compostable packaging in the compostables bin after using it |  |  |
| Putting compostable packaging in the compostables bin benefits the environment |  |  |
| **Automatic motivation** | .64 | .57 |
| Putting compostable packaging in the compostables bin is something that I do without thinking |  |  |
| I’m too busy at work to sort waste |  |  |

*Note.*

Items were informed by previous research (e.g., Allison, Lorencatto, et al., 2022) and were reviewed and refined based on feedback from project partners.

Physical capability was not assessed as it was not considered relevant to assess in this context; Items were displayed in a randomized order.

The items reflecting each of the COM-B components were averaged to create subscales^[[1]](#footnote-1)^.

### **Supplementary Materials 8, text (S8)**

### **Awareness of intervention components**

Most survey respondents reported being aware of the packaging labels (65%, n = 62; not noticed, n = 26; 27%; unsure, n = 7; 7.4%) and bin signage (67%, n = 63; not noticed n = 20; 21%; unsure, n = 11; 12%). However, only 14% (n = 13; not noticed, n = 78; 83%; unsure, n = 3; 3%) reported watching the intervention video. When asked to select which components they believed were most effective at helping participants to put packaging in the compostable bin^[[2]](#footnote-2)^, three participants (4%) selected all three on-site interventions (packaging labels, bin signage and video). Forty-four participants selected both the packaging labels and bin signage (52%), twenty-one selected the bin signage only (25%), fifteen selected the packaging labels only (18%) and one participant selected the video (1%). As such, the bin signage was selected most frequently as the most effective intervention component (n = 65; 77%), followed = 7 by the packaging labels (n = 59; 70%).

**Supplementary Materials 9, Text (S9). Assessing intervention fidelity**

The evidence that we obtained suggested that the interventions were delivered as intended. For labels, workplace C scored high with evidence that all packaging was labelled with the trial’s label. Workplace A and B were not rated as no photos of packaging during the intervention were obtained. For signs, workplace A scored high. Workplace C scored medium because while signs were displayed, some signs had been adapted with changes to the text and images on the signs (the pink color remained consistent). For the video, workplace A and C scored high (circulated via two channels) and workplace B scored medium (circulated via one channel). Of note, workplace A circulated the video via emails from day 1 but the video was not circulated via the screen in the canteen until day 9. Similarly, workplace B circulated the video from day 11. Workplace C circulated the video from day 1. For the on-boarding presentation, workplace A and C scored high (live online presentation delivered) and workplace B scored medium (pre-recorded presentation shared).

**Supplementary Materials 10, Table (S10). Percentage waste and weight (kg) that was compostable packaging or food in the compostables, dry mixed recycling and general waste bins at pre-intervention, post-intervention and follow-up.**

|  | **Pre-intervention** | **~2 weeks^a^** | **Post-intervention** | **Follow-up** | **~6 months^a^** |
| --- | --- | --- | --- | --- | --- |
| **Compostable bins** |  |  |  |  |  |
| *Workplace A* |  |  |  |  |  |
| Compostable packaging | 38% (6.00 kg) | 31% (3.80 kg) | 58% (11.0kg) | 88% (18.08 kg) | 43% (6.5 kg) |
| Food | 8% (1.28 kg) | 10% (1.30 kg) | 13% (2.46 kg) | 1% (0.14 kg) | 5% (0.80 kg) |
| *Workplace B* |  |  |  |  |  |
| Compostable packaging | 25% (3.20 kg) | - | 88% (8.80 kg) | 72% (7.50 kg) | - |
| Food | 5% (0.65 kg) | - | 3% (0.3 kg) | 14% (1.43 kg) | - |
| *Workplace C* |  |  |  |  |  |
| Compostable packaging | 32% (7.02 kg) | 72% (14.30 kg) | 37% (11.10 kg) | 71% (11.05 kg) | 54% (7 kg) |
| Food | 59% (12.9 kg) | 26% (5.27 kg) | 43% (13 kg) | 29% (4.44 kg) | 28% (3.60 kg) |
| **Dry Mixed Recycling** |  |  |  |  |  |
| *Workplace A* |  |  |  |  |  |
| Compostable packaging | 10% (2.45 kg) | - | 0% (0 kg) | 11% (2.73 kg) | - |
| Food | 8% (1.81 kg) | - | 4% (0.80 kg) | 1% (0.35 kg) | - |
| *Workplace B* |  |  |  |  |  |
| Compostable packaging | 10% (1.93 kg) | - | 1% (0.21 kg) | 7% (1.10 kg) | - |
| Food | 3% (0.61 kg) | - | 0% (0 kg) | 9% (1.40 kg) | - |
| *Workplace C* |  |  |  |  |  |
| Compostable packaging | 2% (0.42 kg) | - | 2% (0.30 kg) | 0% (0.05 kg) | - |
| Food | 0% (0.00 kg) | - | 2% (0.40 kg) | 0% (0.00 kg) | - |
| **General waste** |  |  |  |  |  |
| *Workplace A* |  |  |  |  |  |
| Compostable packaging | 2% (0.48 kg) | - | 3% (0.91 kg) | 13% (4.40 kg) | - |
| Food | 14% (3.46 kg) | - | 2% (0.58 kg) | 3% (0.86 kg) | - |
| *Workplace B* |  |  |  |  |  |
| Compostable packaging | 11% (4.24 kg) | - | 6% (2.29 kg) | 7% (2.20 kg) | - |
| Food | 19% (7.33 kg) | - | 0% (0.00 kg) | 11% (3.70 kg) | - |
| *Workplace C* |  |  |  |  |  |
| Compostable packaging | 3% (0.81 kg) | - | 7% (1.10 kg) | 4% (1.44 kg) | - |
| Food | 5% (1.53 kg) | - | 11% (1.60 kg) | 3% (1.04 kg) | - |

*Note.*

^a^Waste assessments conducted at ~two-weeks and ~6 months were additional to the main assessments planned at pre-intervention, post-intervention and follow-up. These additional assessments were conducted on the compostable bins only, not the dry mixed recycling and general wate bins.

Waste assessments were based on a sub-sample of each bin and one week’s collected waste at each time point.

**Supplementary Materials 11, Table (S11). Percentage waste and weight (kg) that was contaminating materials and food in the compostable bins at pre-intervention, post-intervention and follow-up.**

|  | **Pre-intervention** | **~2-weeks^a^** | **Post-intervention** | **Follow-up** | **6-months** |
| --- | --- | --- | --- | --- | --- |
| **Workplace A** |  |  |  |  |  |
| Contamination total^a^ | 51% (8.22 kg) | 59% (8.6 kg) | 30% (5.68 kg) | 12% (2.42 kg) | 52% (8.10 kg) |
| General waste | 27% (4.39 kg) | 47% (5.8 kg) | 0% (0.00 kg) | 0% (0.00 kg) | 15% (2.40 kg) |
| Card | 9% (1.41 kg) | 0% (0.00 kg) | 0% (0.00 kg) | 0% (0.00 kg) | 11% (1.60 kg) |
| Coffee cups | 8% (1.25 kg) | 8% (1.00 kg) | 0% (0.00 kg) | 0% (0.00 kg) | 13% (2.00 kg) |
| Office paper | 2% (0.29) | 0% (0.00 kg) | 0% (0.00 kg) | 0% (0.00 kg) | 8% (1.20 kg) |
| Mixed paper | 1% (0.15) | 0% (0.00 kg) | 0% (0.00 kg) | 0% (0.00 kg) | 0% (0.00 kg) |
| Mixed plastics | 5% (0.73 kg) | 4% (0.5 kg) | 30% (5.68 kg) | 12% (2.42 kg) | 4% (0.70 kg) |
| Food | 8% (1.28 kg) | 10% (1.30 kg) | 13% (2.46 kg) | 1% (0.14 kg) | 5% (0.80 kg) |
|  |  |  |  |  |  |
| **Workplace B** |  |  |  |  |  |
| Contamination total^a^ | 71% (9.06 kg) | - | 10% (1.04 kg) | 14% (1.44 kg) | - |
| General waste | 26% (3.32 kg) | - | 0% (0.00 kg) | 4% (0.4 kg) | - |
| Card | 9% (1.14 kg) | - | 0% (0.00 kg) | 4% (0.41 kg) | - |
| Coffee cups | 3% (0.38 kg) | - | 0% (0.00 kg) | 6% (0.63 kg) | - |
| Office paper | 15% (1.92 kg) | - | 0% (0.00 kg) | 0% (0.00 kg) | - |
| Mixed paper | 0% (0.00 kg) | - | 0% (0.00 kg) | 0% (0.00 kg) | - |
| Mixed plastics | 11% (1.42 kg) | - | 10% (1.04 kg) | 0% (0.00 kg) | - |
| Aluminium ferrous metals | 5% (0.66 kg) | - | 0% (0.00 kg) | 0% (0.00 kg) | - |
| Food | 5% (0.65 kg) | - | 3% (0.3 kg) | 14% (1.43 kg) | - |
|  |  |  |  |  |  |
| **Workplace C** |  |  |  |  |  |
| Contamination total^a^ | 8% (1.77 kg) | 0% (0.00 kg) | 18% (5.5 kg) | 0% (0.00 kg) | 17% (2.40 kg) |
| General waste | 0% (0.00 kg) | 0% (0.00 kg) | 18% (5.5 kg) | 0% (0.00 kg) | 5% (0.70 kg) |
| Card | 0% (0.00 kg) | 0% (0.00 kg) | 0% (0.00 kg) | 0% (0.00 kg) | 6% (0.80 kg) |
| Coffee cups | 0% (0.00 kg) | 0% (0.00 kg) | 0% (0.00 kg) | 0% (0.00 kg) | 1% (0.20 kg) |
| Office paper | 0% (0.00 kg) | 0% (0.00 kg) | 0% (0.00 kg) | 0% (0.00 kg) | 5% (0.70 kg) |
| Mixed paper | 0% (0.00 kg) | 0% (0.00 kg) | 0% (0.00 kg) | 0% (0.00 kg) | 0% (0.00 kg) |
| Mixed plastics | 0% (0.00 kg) | 0% (0.00 kg) | 0% (0.00 kg) | 0% (0.01 kg) | 0% (0.00 kg) |
| Aluminium ferrous metals | 0% (0.00 kg) | 0% (0.00 kg) | 0% (0.00 kg) | 0% (0.00 kg) | 0% (0.00 kg) |
| Fines | 8% (1.77 kg) | 0% (0.00 kg) | 0% (0.00 kg) | 0% (0.00 kg) | 0% (0.00 kg) |
| Food | 59% (12.9 kg) | 26% (5.27 kg) | 43% (13 kg) | 29% (4.44 kg) | 28% (3.60 kg) |

*Note.*

Contamination total did not include food waste as food waste in the compostable bins means food is redirected away from landfill. Food waste in each bin is reported in S12.

**Supplementary Materials 12, Text (S12). A summary of food waste in compostable bins**

Before the intervention, there was a relatively low percentage of food waste in compostable bins at workplaces A (8%) and B (5%), but a relatively high percentage at workplace C (59%). At workplace C, the percentage of waste that was food in compostable bin reduced at post-intervention and follow-up (see S10). For workplace A, the percentage of food waste in compostable bins increased by 5% between pre- and post-intervention and reduced to small amounts by follow-up. There was a 9% increase between pre-intervention and follow-up at workplace B but small changes between pre- and post-intervention.

**Supplementary Materials 13, Table (S13).** Characteristics of survey participants who completed the pre-intervention and post-intervention surveys.

| **Variable** | **n (%) or mean ± SD (95% confidence intervals)** | |
| --- | --- | --- |
|  | **Pre-intervention** | **Post-intervention** |
| **Site**  Workplace A  Workplace B  Workplace C  Unspecified  Total | 32 (38%)  23 (27%)  25 (29%)  1 (6%)  81 | 27 (29%)  48 (52%)  15 (16%)  3 (3%)  93 |
| **Role**  Manager  Office worker/ professional  Cleaner/retail staff  Other  Prefer not to say  Total n | 38 (47%)  32 (40%)  4 (5%)  6 (7%)  1 (1%)  81 | 37 (40%)  45 (48%)  10 (11%)  0 (0%)  92 |
| **Canteen use frequency**  Once a year  2-3 times a year  Every two months  Once a month  2-3 times a month  Once a week  2-3 times a week  4-6 times a week  Daily  Total n | 0 (0%)  1 (1%)  0 (0%)  0 (0%)  6 (7%)  18 (22%)  36 (44%)  10 (12%)  10 (12%)  81 | 1 (1%)  2 (2%)  1 (1%)  6 (6%)  10 (11%)  13 (14%)  40 (43%)  7 (8%)  13 (14%)  93 |
| **Duration worked at site**  Unspecified/unclear  Up to 6 months  6 months – <1 year  ≥ 1 year; ≤ 5 years  ≥ 5 years  Total n^a^ | 11 (14%)  4 (5%)  7 (9%)  21 (26%)  38 (47%)  81 | 12 (13%)  8 (9%)  8 (9%)  31 (34%)  31 (34%)  90 |
| **Gender**  Male  Female  Prefer not to say  Total n | 32 (40%)  47 (58%)  2 (2%)  81 | 24 (26%)  67 (72%)  2 (2%)  93 |
| Age^b^ | 41.6 ± 12.0 (38.9, 44.3) | 39.3 ± 11.6 (36.9, 41.7) |
| Completed both surveys?^c^ |  | Yes: 22 (24%)  No: 60 (62%)  Unsure: 15 (14%) |

*Note.*

Dropped out of the survey before completing demographic questions: pre-intervention n = 4; Post-intervention, n = 4.

^a^Post-intervention, three participants did not provide a response.

^b^Pre-intervention age n = 78 (no response n = 2; implausible value which was removed, n = 1); post-intervention age n = 89 (no response n = 3; implausible value which was removed, n = 1).

^c^Participants’ who responded to the pre- and post-intervention surveys were of a similar age [t(165) = 1.24, *p* = .22] and the proportion of men and women respondents did not significantly vary at pre- and post-intervention [χ²(1) = 3.82, *p* = .05)].

**Supplementary Materials 14, Table (S14). Acceptability of the intervention for employees and workplace leads.**

| **Construct** | **n (%) or Mean ± SD (95% CI)** |
| --- | --- |
| **Acceptability (Employees survey n = 93)** |  |
| Affective attitude | 4.10 ± 0.75 (3.94, 4.25) |
| Perceived effectiveness | 3.75 ± 0.93 (3.55, 3.94) |
| Intervention coherence | 3.88 ± 0.97 (3.68, 4.08) |
| Self-efficacy^a^ | 3.91 ± 0.93 (3.72, 4.10) |
| Opportunity costs | 1.63 ± 0.70 (1.49, 1.78) |
| Burden (effort) | 1.70 ± 0.79 (1.54, 1.86) |
| General acceptability | 4.24 ± 0.79 (4.07, 4.40) |
| **Acceptability (Workplace leads survey, n = 9)** |  |
| Affective attitude | 4.44 ± 0.53 (4.04, 4.85) |
| Perceived effectiveness | 3.67 ± 0.87 (3.00, 4.33) |
| Intervention coherence | 4.00 ± 0.50 (3.61, 4.38) |
| Self-efficacy | 4.33 ± 0.50 (3.95, 4.72) |
| Opportunity costs | 2.78 ± 1.30 (1.78, 3.78) |
| Burden (effort) | 3.22 ± 0.83 (2.58, 3.86) |
| Ethical considerations | 3.11 ± 0.93 (2.40, 3.82) |
| General acceptability | 4.56 ± 0.53 (4.15, 4.96) |

*Note.*

Intervention acceptability was measured with the Theoretical Framework of Acceptability (Sekhon et al., 2017; Sekhon et al., 2022).

^a^Self-efficacy was measured at both pre-intervention and post-intervention. At pre-intervention mean scores were 3.89 ± 0.86 (n = 82).

Possible scores ranged between 1-5 with higher scores indicating greater acceptability, except for opportunity costs, burden and ethical considerations, whereby lower scores indicate greater acceptability.

**Supplementary Materials 15, Table (S15). Workplace Leads’ ratings of the intervention components (frequencies) collected during the roundtable event.**

| Component | Not at all useful | Not useful | No opinion | Useful | Extremely useful |
| --- | --- | --- | --- | --- | --- |
| Packaging labels | 0 | 1 | 0 | 3 | 5 |
| Signs | 0 | 0 | 1 | 5 | 3 |
| Video | 0 | 0 | 1 | 4 | 4 |
| Presentation | 0 | 0 | 2 | 3 | 4 |
| **Open-ended explanations for ratings:** | | | | | |
| **Packaging labels** | | | | | |
| *Great feedback from staff who found it easy and a time saver* | | | | | |
| *Again the colour coding was the major win, it helps as a quick identifier when throwing waste in the bins.* | | | | | |
| *Clarity and bright colours* | | | | | |
| *A lot clearer than previous signage* | | | | | |
| *I think it helped people to identify which items were compostable* | | | | | |
| *Very helpful. , colour really help* | | | | | |
| *Better guidance for people in the offices* | | | | | |
| *I think they are bit too small* | | | | | |
| **Signs** | | | | | |
| *Shows what products need to compost* | | | | | |
| *Clear to avoid confusion.* | | | | | |
| *Made it clearer what is compostable* | | | | | |
| *Visual images of the compostable packages* | | | | | |
| *I don’t think this was visible* | | | | | |
| **Video** | | | | | |
| *Quick way to educate people* | | | | | |
| *We played the video on a loop in our staff canteen, which allowed for maximum coverage.* | | | | | |
| *Engaging and informative* | | | | | |
| *Have shared for other (redacted) contracts* | | | | | |
| *Easy to understand* | | | | | |
| *Really well explained and engaging video* | | | | | |
| **On-boarding presentation** | | | | | |
| *Easy to understand the aims objectives and benefits* | | | | | |
| *It helped with our comma strategy* | | | | | |
| *Great knowledge shared* | | | | | |
| *Needed to understand the project* | | | | | |
| *I think for us we had the presentation once we had implemented the trials* | | | | | |

**Supplementary Materials 16, Text and Table (S16).** **Summary of main themes generated in the roundtable event with workplace leads.**

Five main themes were identified by researchers from the roundtable discussions. All are listed in the table below. Three main themes are discussed below and summarised in the table below.

**Perceived effectiveness of intervention components**

The pink color on signage and labels was considered the most effective component of the intervention. Workplaces discussed how the color was different to other bins and that this disrupted the automatic behavior to put waste in bins. Others said how the signs led employees to actively look for the appropriate bin to use. One workplace said that the distinctive color addressed the barrier that compostable packaging looks like plastic.

**Impacts on waste management practices**

One workplace said that supplying compostable packaging alone was insufficient and that additional approaches, such as the intervention components, were needed. The same workplace was keen to continue applying labels and were encouraging client organizations to implement the intervention components. The pros and cons of using workplace specific labels (non-standardized) was discussed; noting that, without external regulation, labels could be inappropriately used on non-certified compostable packaging items. Another workplace said they would consider continuing to apply labels, however, the other workplace raised concerns over the feasibility of applying labels in-house.

Some workplaces reported that the trial had impacted wider waste management practices, such as introducing food caddies (C) and increased collections for compostable packaging (A). Workplace C said they planned to use the trial to inform upcoming changes to other waste streams. Workplaces also noted how they were already engaging in sustainable waste management practices.

**Remaining barriers**

Confusion understanding the term ‘compostable packaging’ was raised as a remaining barrier after the intervention. Workplaces also discussed the challenge around having multiple brands of compostable packaging, each having varying labels. Although another workplace felt that the relatively small number of compostable packaging brands facilitated sorting waste. One workplace referred to a high turnover of staff and people working from home as a reason for continued challenges with sorting waste.

| **Theme**  *(sub-themes)* | **Summary of discussion points with quotes** |
| --- | --- |
| **Perceived effectiveness of intervention components** | |
| *Pink colour for signs and label* | - Beliefs that the pink labels, signs or colour coding likely had the most impact – ‘*They were the biggest difference-maker*’ - The colour was distinctive to other bins which disrupted the automatic sorting of waste and was especially useful when where were multiple bins. - Employees were actively searching for the appropriate bin - Addressed the barrier that compostable packaging looks like plastic. - Leads reported receiving positive feedback from employees about the label (“*One of our people who hates change…sent me a photo of this sticker and said, 'This is amazing.'*”). |
| *Video* | - Covered the motivating reasons for composting – ‘*there is a point to it as well, it's not just put it in the bin and then this is what happens, this is the good bit*’ |
| **Impacts on waste management practices** | |
| *Compostable packaging alone is insufficient* | - Supplying compostable packaging helped workplaces to feel they were doing the right thing. However, one workplace acknowledged that more was needed to ensure people sort and dispose of compostable packaging appropriately. |
| *Continuing with labels on compostable packaging* | - There were mixed responses around continuing applying labels to compostable packaging.   - One workplace was keen to continue and encourage other workplaces they work with to implement all intervention components. This workplace discussed the potential to purchase labels and have catering teams apply stickers.   - One workplace had not yet considered continuing use of labels but said they would think about.   - The other workplace raised concerns around the labour intensity of applying labels. |
| *Changes to other waste streams* | - One workplace reported on more regular waste collections for compostable bins. - One workplace has introduced food caddies to reduce the amount of food in the compostable bins. - Review and refresh of all waste streams (e.g. recycling) based on the trial - ‘*We were due a big refresh of all our recycling…Let’s wait till this has finished, see if there’s anything we’ve learnt,’ that we can go forwards.*’ - Change wording used at bins across the waste streams - (‘*We’re going to change our bins to try and maybe simplify that down a little bit more… when we did our bins originally, I don’t know, maybe we tried to make it a bit quirky, whereas now, we’re just going to go to bullet points, ‘This is what goes in the bin*’) - However, one workplace said they are already taking action with other streams but would use the trial to build on their efforts (“*we already, kind of, try and do-, we've recently, this year, been doing a lot more promotion around recycling and what goes in which bin and how we can try and improve that. So, I think we can use parts of this trial to, kind of, build upon”*. The other site also said they are actively engaged in efforts to improve waste behaviours and listed several initiatives they are engaged in - ‘*we’ve also got, like, champions, sustainability champions and things. That’s not our head office, that’s for all of our just general waste and things. So, there, we have, you know, like a leader-board and things like that to keep people engaged.*’ |
| **Remaining barriers** |  |
| *Confusion with the term ‘compostable packaging’* | - Confusion with the term compostable packaging and confusing industrial and home composting. - At one workplace, one person brought in their food waste from home. |
| *Variety of compostable packaging brands* | - People can recognise ANONYMIZED (packaging producer) but identifying compostable packaging is made difficult by the availability of a range of brands supplying compostable plastic packaging – ‘*the other thing that was discussed internally as well is that obviously,* ANONYMIZED (packaging producer)*, it's* ANONYMIZED (packaging producer) *but it's not the only compostable.*’ - One workplace referred to having limited brands of compostable packaging as a facilitator for appropriate waste sorting – ‘*we don't have a lot of different options for disposables so actually, that's kind of helped with that messaging because they understand the fact that it is* ANONYMIZED (packaging producer) *so nine times out of ten, it's going in that bin.’* |
| *Issues with waste segregation* | - One workplace referred to remaining issues with waste sorting due to high staff turnover and staff not regularly in the office who were not exposed to the intervention components – ‘*we did struggle in the main office with people still not segregating, you've got, like, a high turnover of staff and people who don't come into the office regularly*’ |
| **Ways to develop and improve the intervention** | - One workplace said it would have been useful to receive feedback on their site’s waste performance sooner so it could be fed back to the team to motivate further effective waste sorting. - Another workplace agreed on the value of feedback and that it can be useful to for competition within workplaces (not between workplaces) |
| **Intervention fidelity** | - Workplaces commented on displaying the video for 4-weeks and via multiple channels. However, some comments indicated some deviations from delivering the interventions as intended. One workplace said it had not been possible to play the video with sound. - Workplace C also indicated that further communications had been sent out regarding the labels than planned ‘*we send a message out if it's got a coloured sticker on it, it goes in that bin and that was very easy for people to just, kind of, almost like, 'Okay, that's fine.' So the minute they received it, they just knew and it went in. We found actually that seemed to be the winner*.’ |

1. Responses with missing items were included in the average provided a sufficient number of items had been completed per component. Psychological capability, reflective motivation and physical opportunity ≥ three items completed; Social opportunity and automatic motivation ≥ two items completed. At pre-intervention, Scores computed based on incomplete scale responses: pre-intervention n = 9; post-intervention n = 2. [↑](#footnote-ref-1)
2. This question was completed by 84 participants. [↑](#footnote-ref-2)
